# Supplementary material for: The contributions of entorhinal cortex and hippocampus to error driven learning
Source: Commun Biol. 2021 May 24;4:618. doi: 10.1038/s42003-021-02096-z (PMC8144598; doi:10.1038/s42003-021-02096-z)
Supplement: Supplementary file 2 — supplementary information [file 42003_2021_2096_MOESM2_ESM.pdf]

Supplementary Information

The contributions of entorhinal cortex and hippocampus to error driven learning

Shih-pi Ku, Eric L. Hargreaves, Sylvia Wirth, and Wendy A. Suzuki

Correspondence:

Shih-pi Ku shihpi@gmail.com

Wendy A. Suzuki ws21@nyu.edu

Supplementary figure 1

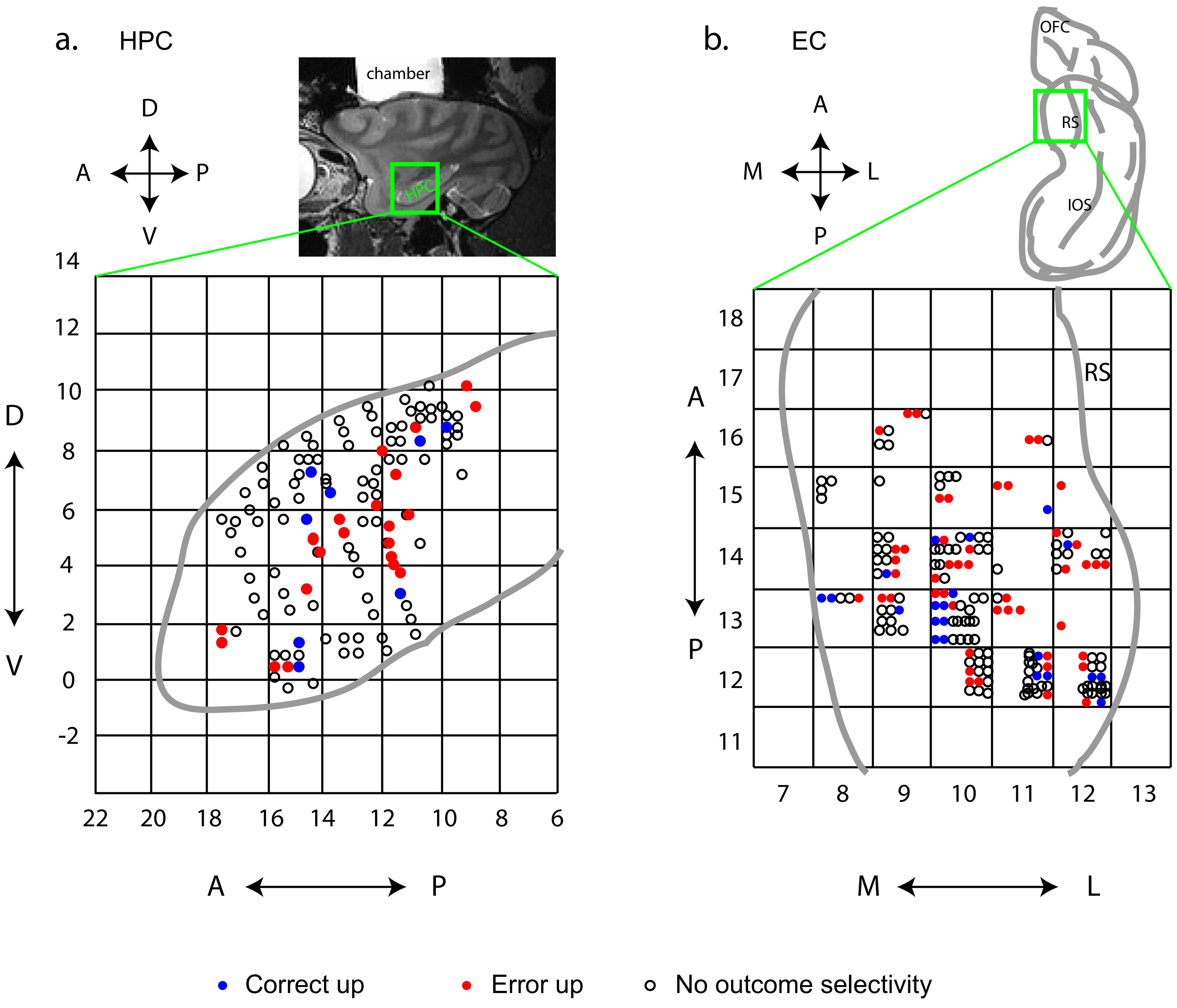

Supplementary Figure 1 Recording sites. a) Sagittal MRI image at the top of figure is of monkey El with the hippocampus (HPC) outlined by a green box and the location of the recording chamber indicated by the bright area dorsal to the brain. The graph at the bottom shows an expanded version of the same sagittal view of the HPC shown in the MRI image illustrating the anterior-posterior and dorsal ventral coordinates of the recording sites for monkeys A, B and El. Because we did not observe any clear topography, we compressed all the medial-lateral recording sites. The thick gray line illustrates the outline of the sagittal view of the anterior 2/3 of the HPC. The X- and Y- axes show the stereotaxic coordinates in mm from the crossing point of the interaural line and mid-sagittal line. Blue dots indicate the correct up cells, red dots indicate the error up cells and the open circles indicate the non-outcome selective cells. **b)** Line drawing at the top of the figure illustrates a ventral view of a monkey brain showing the location of the entorhinal cortex (green box). The graph below shows an expanded version of the EC illustrating the anterior-posterior and medial-lateral coordination of the recording sites in monkeys A and B. The same as in panel A, the X- and Y axes of the graph indicate the stereotaxic coordinates in mm from the crossing point of the interaural line and mid-sagittal line. No clear topography was observed. Abbreviations: A: anterior; D: dorsal; L: lateral; M: medial; P: posterior; V: ventral. RS: rhinal sulcus; OFC: orbitofrontal cortex; IOS: inferior occipital sulcus.

Supplementary figure 2

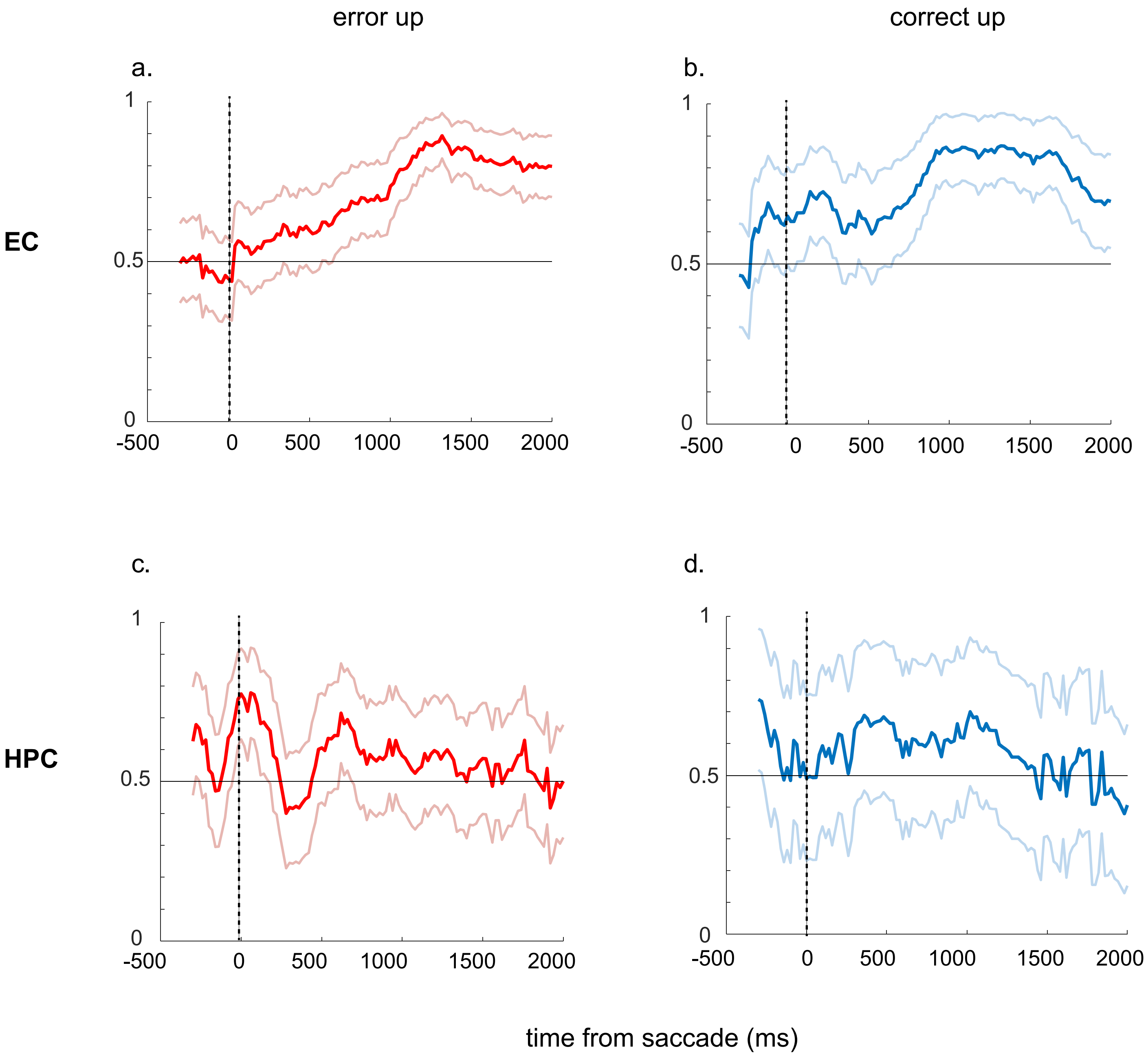

Supplementary Figure 2 Time courses of area under Receiver operating characteristic curves. The averaged time courses of the area under ROC curve of error-up (a) and correct-up cells (b) in entorhinal cortex (EC) and the error-up (c) and correct-up cells (d) in hippocampus (HPC) are plotted to show the strength of each category of outcome selective neurons in differentiating correct from error outcomes. The lighter lines indicate the standard error of the mean of the averaged time courses.

Supplementary figure 3

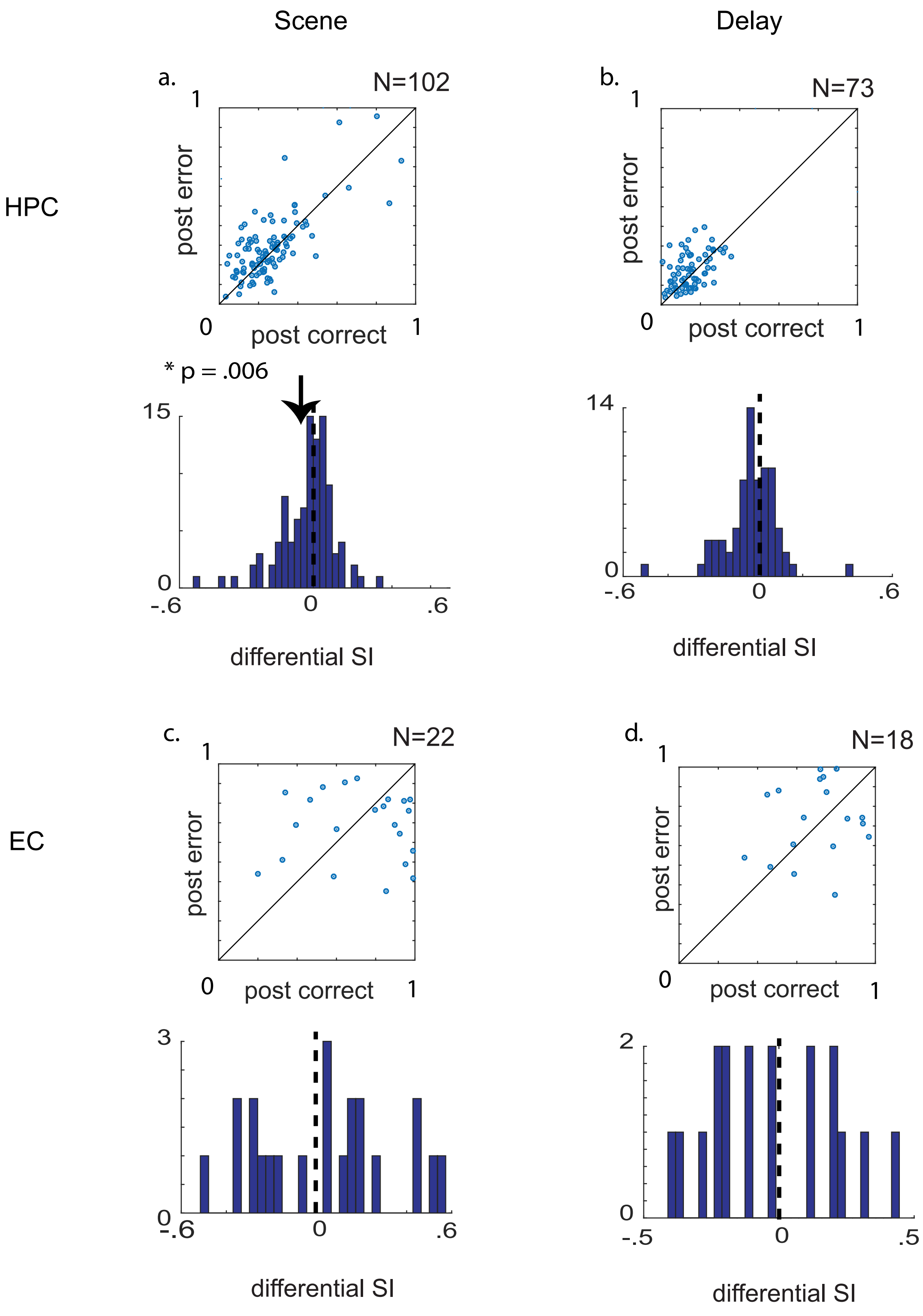

Supplementary Figure 3 The differential selectivity (SI) of hippocampal neurons whose selectivity index surpassed the threshold obtained by permutation test was significantly different from zero during scene presentation period in LST task. The SI of hippocampal neurons, whose SI surpassed the permutation test threshold during scene were plotted in (A) and delay in (B). The SI of entorhinal neurons, whose SI surpassed the permutation test threshold during scene were plotted in (C) and delay in (D).

Supplementary figure 4

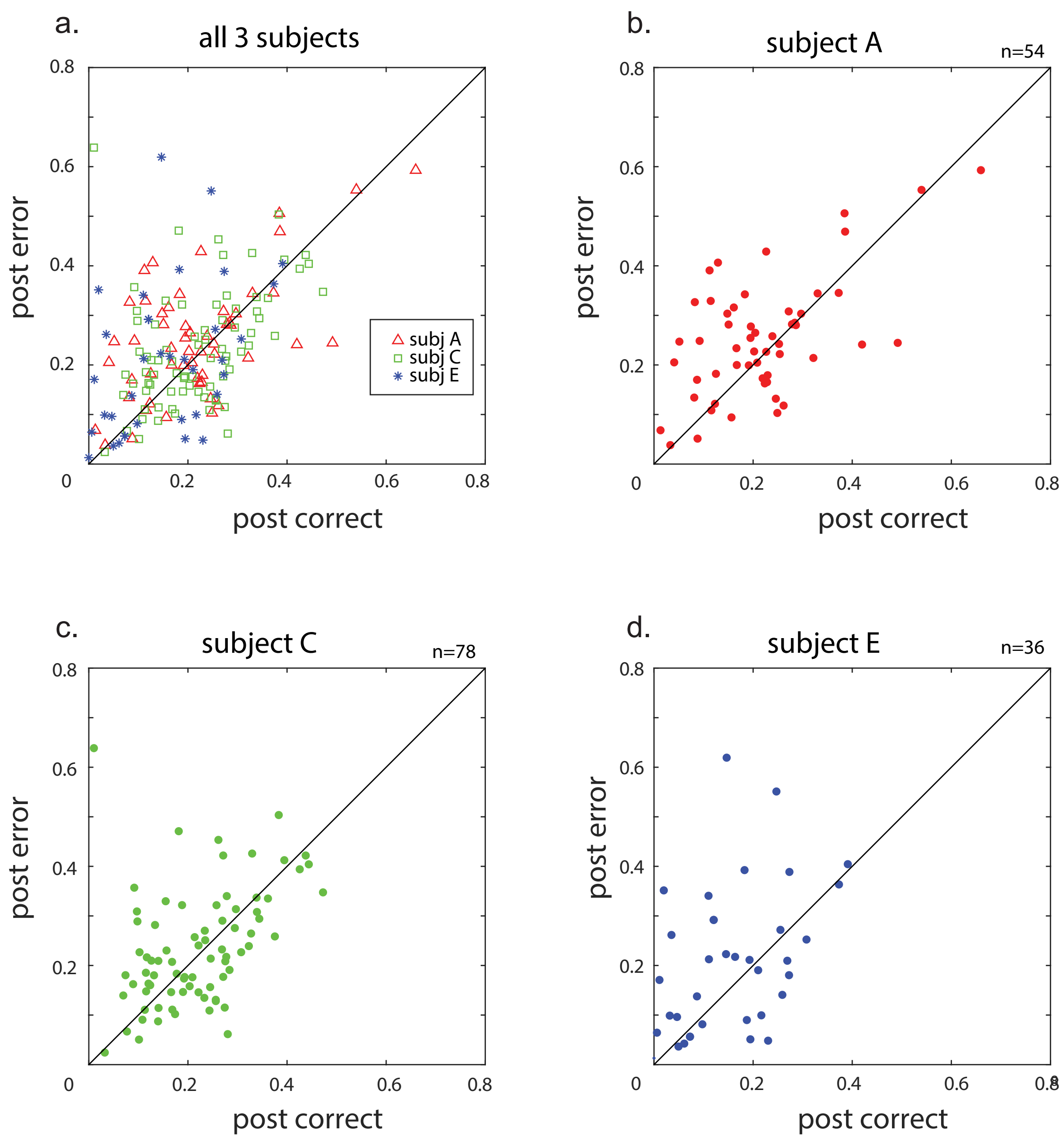

Supplementary Figure 4 Hippocampal selectivity of individual animals for scenes during early learning stage of LST, showing no dependency of different subjects/macaques in variance analysis ($X^2(166) = 45.2$, p <.001 for post-correct SI and $X^2(166) = 45.1$, p < ,001 for post-error SI, *Chi*-square test of independence). a) Overlaying the selectivity indices of all subjects. b) Showing the SI of subject A only. c) Showing the subject C only. d) Showing the subject El only.

Supplementary Figure 5

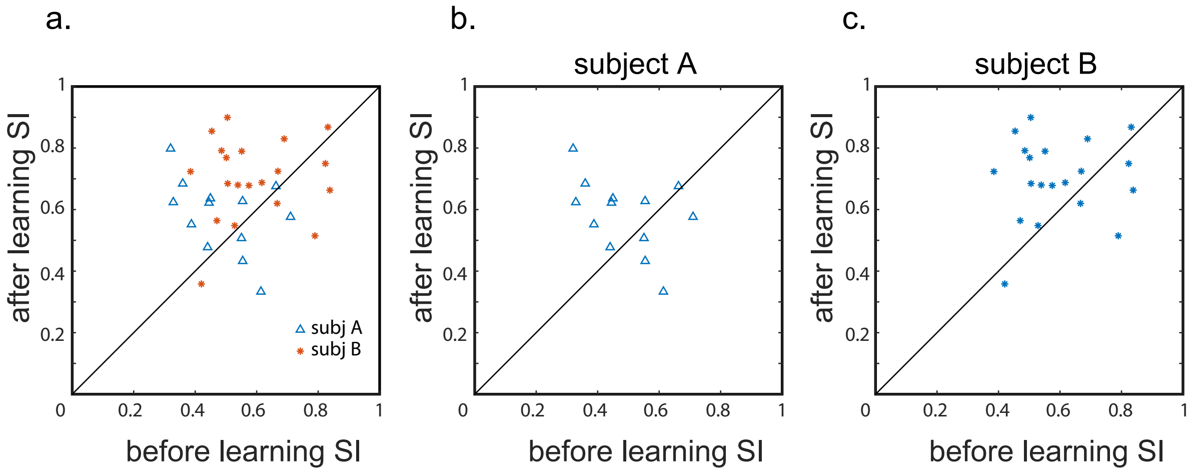

Supplementary Figure 5 Entorhinal selectivity of individual animals for delay period comparing before versus after learning stage of LST, showing no dependency of different subjects in variance analysis ($X^2(126) = 6.23$, p <.001 for post-correct SI and $X^2(126) = 7.68$, p < ,001 for post-error SI, *Chi*-square test of independence). A) Overlaying the selectivity indices of both subjects. B) Showing the SI of subject A only. C) Showing the subject B only.

Supplementary fig. 6

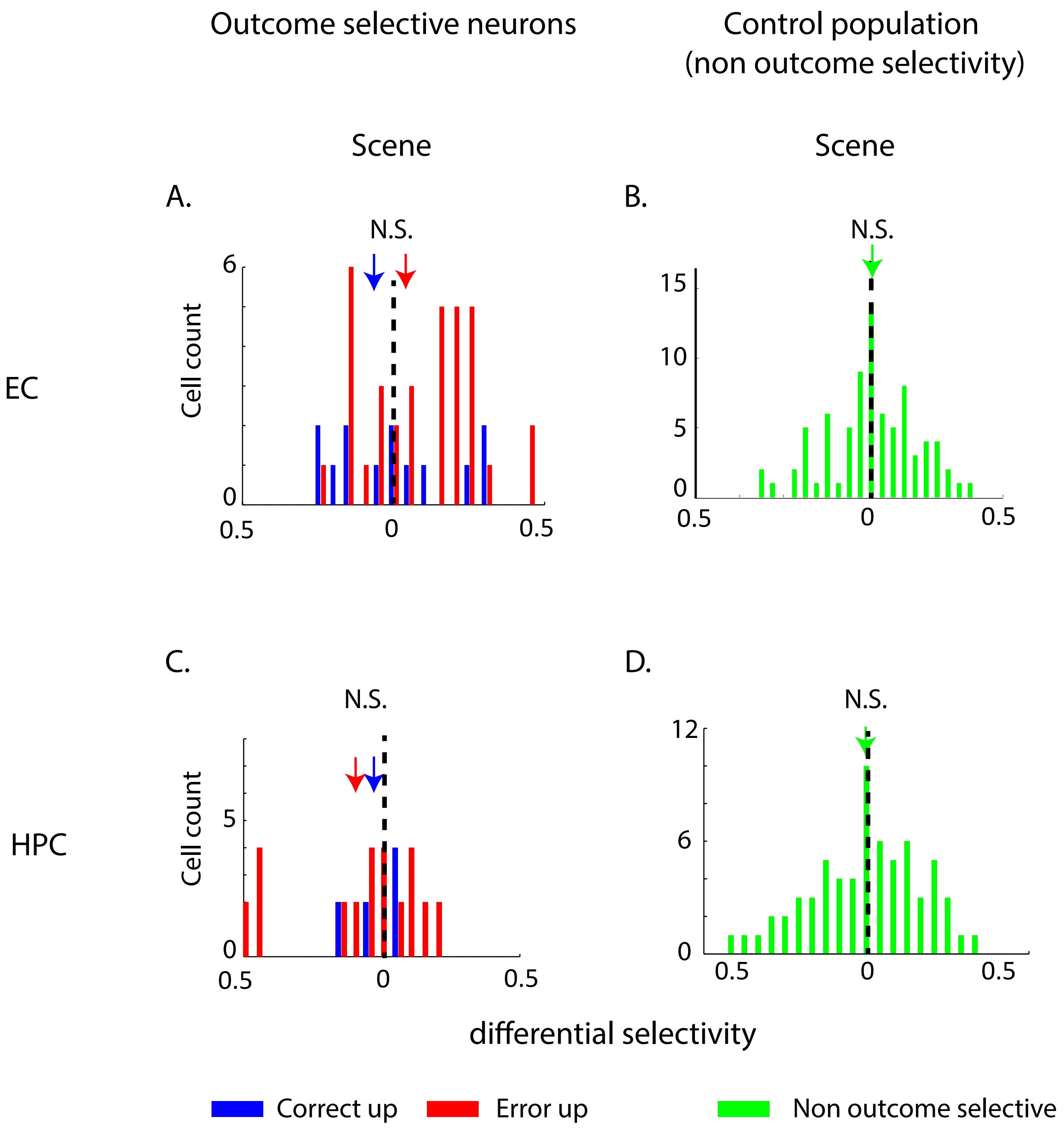

Supplementary Figure 6 Differential Selectivity indices (SI). The differential selectivity indices of the correct up and error up cells in EC and HPC during scene period are plotted in sup. fig. 6a and c. The differential selectivity indexes of the non-outcome selective cells in EC and HPC during scene and delay period are plotted in sup. fig. 6b and d. N.S.: not significant.

Supplementary fig. 7

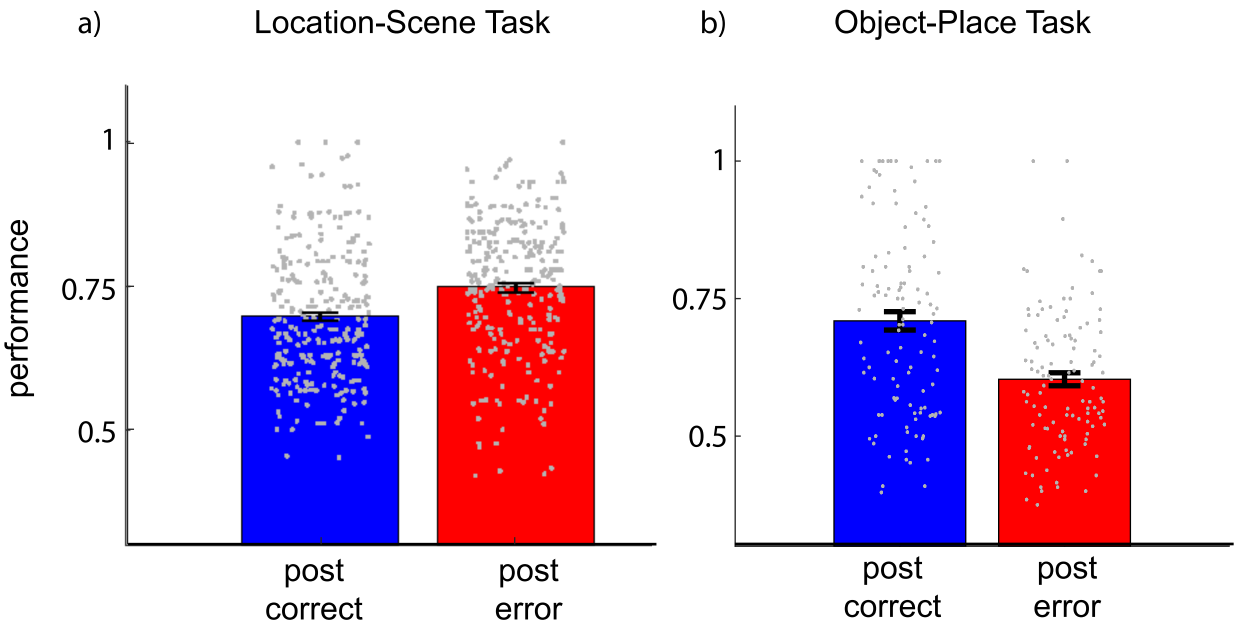

Supplementary Figure 7 All the datapoints of performances were overlaid on the bar plots showing the averaged performance of the Location-Scene Task (a) and the Object-Place Task (b).

Supplementary Table 1

HPC		paired t-test t	dof	p
scene	cor up	2,8978	3	.0626
	err up	1,2576	15	.2278
	no sel	0,7754	111	.4397
delay	cor up	1,0597	3	.3671
	err up	0,1226	15	.9041
	no sel	0,1254	109	.9005

Supplementary Table 1 In hippocampus, neither the error up, correct up nor the non-outcome selective cells changed their selectivity index with learning by comparing the selectivity index before versus after learning.

Abbreviations: HPC: hippocampus, dof: degree of freedom, cor up: correct up cells, err up: error up cells, no sel: no outcome selective cells.
